# Mechanical and Thermal Properties of Polyether Polytriazole Elastomers Formed by Click-Chemical Reaction Curing Glycidyl Azide Polymer

**DOI:** 10.3390/molecules25081988

**Published:** 2020-04-23

**Authors:** Liming He, Jun Zhou, Yutao Wang, Zhongliang Ma, Chunlin Chen

**Affiliations:** 1 School of Environment and Safety Engineering, North University of China, Taiyuan 030051, China; 18334788227@163.com (J.Z.); a1506672228@163.com (Y.W.); ma19960512@sohu.com (Z.M.); 2 Luzhou North Chemical Industries Co., Ltd., Luzhou 646000, Sichuan, China; chenchunlin255@163.com

**Keywords:** click chemical reaction, PTPET, GAP, polytriazole, thermo-mechanical properties

## Abstract

Energetic binders are a research hot-spot, and much emphasis has been placed on their mechanical properties. In this study, propargyl-terminated ethylene oxide-tetrahydrofuran copolymer (PTPET) was synthesized. Then, PTPET and low-molecular-weight ester-terminated glycidyl azide polymer (GAP) were reacted by the click reaction without using catalysts to obtain a polyether polytriazole elastomer. Through tensile tests, where R = 0.5, the tensile strength reached 0.332 MPa, with an elongation at break of 897.1%. Swelling tests were used to measure the cross-linked network and showed that the cross-linked network regularity was reduced as R increased. The same conclusions were confirmed by dynamic mechanical analysis (DMA). In DMA curves, Tg was around −70 to −65 °C, and a small amount of crystallization appeared at between −50 and −30 °C, because locally ordered structures were also present in random copolymers, thereby forming localized crystals. Their thermal performance was tested by Differential Scanning Calorimeter (DSC) and Thermal Gravimetric Analyzer (TG), and the main mass loss occurred at around 350 to 450 °C, which meant that they were stable. In conclusion, the polyether polytriazole elastomer can be used as a binder in a composite propellant.

## 1. Introduction

Exploring the universe is a human dream, and propellant is the power source of spacecraft. The binder is an important component of solid propellant, and attempts have been made to incorporate energetic components into the binder to impart energy to the binder while ensuring good mechanical properties to meet the higher requirements for binders [1,2,3,4,5,6,7,8].

Glycidyl azide polymer (GAP) can be added to composite solid propellants as an energetic component due to its high heat and density; however, in composite propellant binders, conventional curing methods utilize isocyanates as a curing agent for GAP. There are many disadvantages of this curing system: many side reactions, high toxicity, high activity of isocyanate, and sensitivity to moisture, affecting mechanical properties by reacting rapidly with water to form a urea group while releasing carbon dioxide, which can stay inside the propellant. Moreover, high-temperature curing readily causes product defects [9,10]. By contrast, click chemistry, as a simple, efficient, controllable, and fast synthesis method, has attracted wide attention since Kolb et al. proposed the concept of click chemistry in 2001 [11]. Compared with a hydroxy and isocyanate curing system, the alkynyl azide curing system has no side effects, is not sensitive to moisture, has greater thermal stability, and good compatibility with high-energy components (e.g., ammonium dinitramide, hydrazinium nitroformate) which are helpful for the practical application of high-energy components. [1,2,3,5,6,7,12,13].

When using click chemistry, scholars have tried a range of cross-linking agents, such as bis-propargyl-succinate (BPS), aromatic diyne (1,4-diethynylbenzene, 1,4-diacetylenecarbonylbenzene and 1,3-dicyanoethynylbenzene) [14], propargyltriazine (TPC) [15], and propargyl-terminated polyethylene glycol (PEG) [4]. In short, BPS is the preferred curing cross-linker [2,16,17]. According to the literature, due to the rapid reaction of BPS and GAP, it readily forms bubbles, depressions, and other defects; high modulus and low elongation (< 100%) also limit its further use. Herein, to improve the mechanical properties, we replaced BPS with alkynyl-terminated ethylene oxide-tetrahydrofuran copolymer (PTPET), forming GAP-crosslinked propargyl-terminated ethylene oxide-tetrahydrofuran copolymer (PTPET) polyether polytriazole elastomer with GAP. It is hoped that PTPET and GAP will meet the demands of energy performance and mechanical properties, after curing using click chemistry.

## 2. Results and Discussion

### 2.1. Structural Characterisation of PTPET

**Figure 1:**
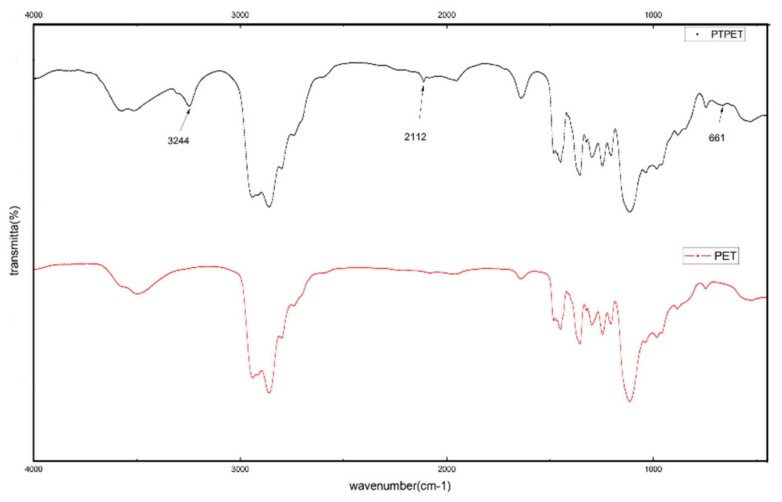
FTIR spectra of PET and PTPET. PET, ethylene oxide-tetrahydrofuran copolymer; PTPET, propargyl-terminated ethylene oxide-tetrahydrofuran copolymer.

The FTIR spectra of ethylene oxide-tetrahydrofuran copolymer (PET) and PTPET are shown in Figure 1. In comparison with the PET spectrum, the PTPET spectrum shows three characteristic peaks of the alkynyl group, which are the characteristic peaks of ≡C-H at 3244 cm^−1^, C≡C at 2112 cm^−1^, and ≡C-H at 661cm^−1^. As a result of the PTPET backbone being a random copolymer of ethylene oxide and tetrahydrofuran, the two ends of the chain are asymmetric, so the absorption peaks of the alkynyl groups do not disappear.

**Figure 2:**
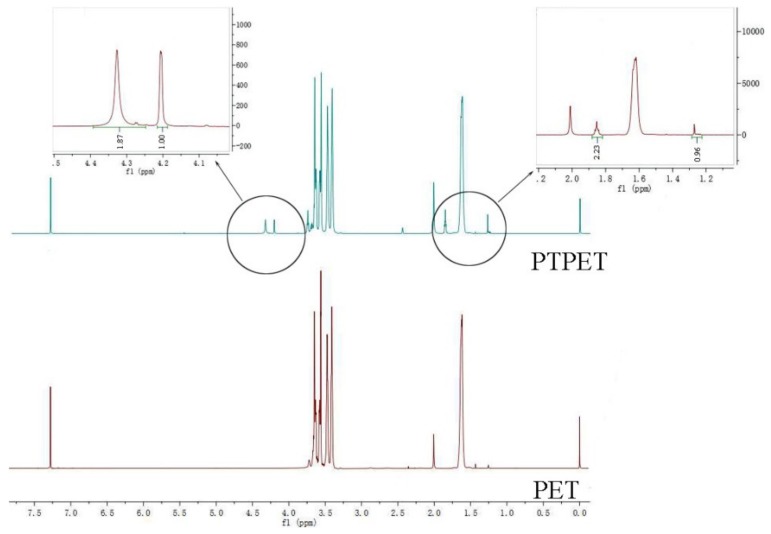
^1^H NMR spectra of PET and PTPET.

The ^1^H NMR spectra of PET and PTPET are shown in Figure 2. From the ^1^H NMR, there are four peaks that can be divided into two groups in the approximate ratio of 1:2. This can be accounted for by the ratios of H on propargyl (-CH_2_-C≡CH) on -CH_2_- and -C≡CH. These indicate that the terminated hydroxyl has been replaced by propargyl, yielding the desired product: PTPET.

### 2.2. Mechanical Properties

**Figure 3:**
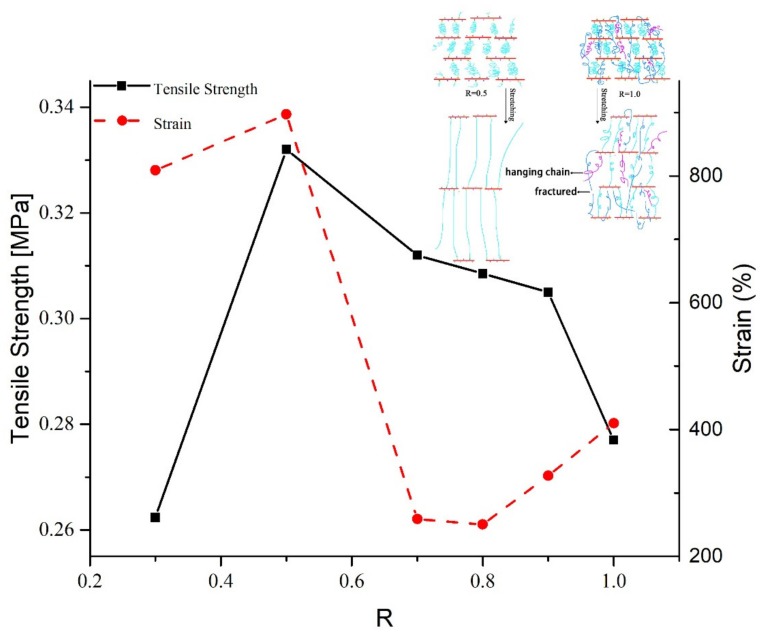
Mechanical properties: effect of R value for polytriazole elastomers S0–S5.

The tensile strength and elongation of samples S0–S5 as a function of the R value are shown in Figure 3 with the mechanical properties of polytriazole elastomers listed in Table 1. As can be seen from the figure, the maximum tensile strength can reach 0.332 MPa. The tensile strength has a minimum value of R = 0.3, and then the tensile strength gradually decreases as R increases [1,4,12,13,17]. The regularity of the crosslinked network affects the mechanical properties of the polymer material. The reason for this phenomenon may be the molecular structure of GAP; because the number of atoms carried by the main chain interval in the molecular structure of GAP is small and a large -CH_2_N_3_ side chain is present, the intermolecular force is small [18]. Due to steric hindrance, when the R value is small, the alkynyl groups freely react with the azide groups on the GAP chain, in other words, steric hindrance can play a role in regulating the cross-linked network. When the R value is bigger, a competition arises and the active alkynyl groups preferentially reacts with the azide groups. After that, the unreacted alkynyl groups need to react with the remaining -N_3_ in a limited space, which will destroy the original regularity. Moreover, the regularity will be further destroyed by a formulate triazole ring. Hence, the bigger the R value, the more irregular the cross-linked network. At R = 0.3, because the number of alkynyl groups is too small, and the number of cross-linking points of the cross-linking network that can be formed is also too small, its tensile strength is very low and the elongation is also low. The parameters of the network structure will be discussed later.

The elongations are all above 200%, and some even approach 900%, showing excellent deformability. The elongation first decreases as the R increases, and has a minimum value at R = 0.8 (thereafter, the elongation increases). A maximum elongation, 897.1%, occurs at R = 0.5, a value far greater than that of other samples. When the R value is small, because there are fewer cross-linking points inside the sample, and the network structure is more regular, the contribution to elongation mainly derives from the orientation of the molecular chain of PTPET, scilicet changing from curled to stretched. However, as the R value increases, the number of cross-linking points increases. Physical entanglement and chemical entanglement between polymer chains decrease the elongation, albeit later it increases because a few cross-linking points in an irregular network may be destroyed preferentially during stretching, making the network more regular again and thus increasing elongation. In addition, as R increases, as a result of the greater apparent average molecular weight M_c_ (Table 2), the tensile strain is increased [19], the contribution of the number of triazole rings increases, and thus the elongation also increases, and the strain at break is increased.

Considering the comprehensive mechanical properties of PTPET, it can be found that when R = 0.5, the strongest tensile strength and a much better elongation can be obtained. In general, the use of PTPET is better than BPS, especially in terms of elongation. However, the tensile strength of elastomers is poor; thus, we need to continue to study methods to improve the tensile strength, for example, through the use of larger molecular weight azide-terminated glycidyl azide polymer (GAPA), or the introduction of a second network.

### 2.3. Network Structure

**Figure 4:**
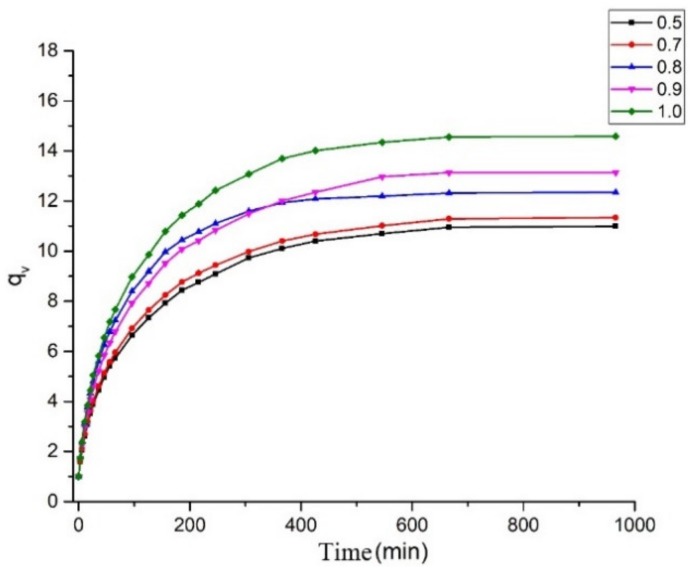
Volume swelling curves of S1–S5.

The volume swelling curves of the polytriazole elastomers are shown in Figure 4. The parameters of the network structure are listed in Table 2. Initially, the slopes of the volume expansion curves are steep and the swelling rates are high. In time, the swelling rates gradually decrease, and by 700 min, q_v_ remains substantially unchanged. As can be seen from the curve, as R increases, q_v_ also increases. In Figure 4, there is a crossover between sample S3 and sample S4. The reason is that sample S3 has a little crack, which causes the early swelling rate to be greater than sample S4.

The equilibrium swelling ratios (q_v_) of the elastomers can be calculated by assuming the additivity of volumes, using Equation (1). Based on the value of q_v_ of polymers, the volume fractions (V_2m_) in the swollen elastomers can be calculated using Equation (2). The Flory-Huggins interaction parameter between the polymer and solvent (χ_1_) can also be evaluated by the Bristow-Watson equation, Equation (3). Thus, the apparent number of average molecular weights (M_c_) of elastomer strands can be further evaluated according to the Flory-Huggins theory (Equation (4)). Furthermore, the apparent cross-linking densities (N_0_) also can be determined by using Equation (5).

(1) $$q_{v}=1+(\frac{w}{w_{0}}-1)\frac{\rho_{2}}{\rho_{1}},$$

(2) $$V_{2m}=\frac{1}{q_{v}},$$

(3) $$\chi_{1}=0.34+V(\delta_{s}-\delta_{p}{)}^{2}/\mathrm{RT},$$

(4) $$M_{c}=-V\rho (V_{2m}^{\frac{1}{3}}-\frac{V_{2m}}{2})/[\ln\left(1-V_{2m}\right)+V_{2m}+\chi_{1}V_{2m}{}^{2},$$

(5) $$N_{0}=\rho /M_{c},$$

where w_0_ is the mass of the sample before swelling, w represents the mass of the specimen after swelling, and ρ_1_ and ρ_2_ are the densities of the solvent and the polymer, respectively, V is the molar volume of toluene solvent (106.4 cm^3^ **·** mol^−1^), R is the gas constant, T is the absolute temperature, ρ denotes the density of the elastomer, and δ_S_ and δ_P_ are the solubility parameters of the elastomer and the solvent, respectively, which are found to be 19.2 (J **·** cm^−3^)^1/2^ and 18.2 (J **·** cm^−3^)^1/2^ [20]. The calculated structural parameters of PTPET-based elastomers are also listed in Table 2.

For samples S1–S5, Mc increases from 7201.1 to 10,416.5 g mol^−1^, and N_0_ decreases from 0.143 to 0.102 mmol cm^−3^ with increasing R value. For the cross-linked elastomer, the stress and modulus are proportional to the density of network chains N_0_, and the rate of migration of the solvent within is inversely proportional to N_0_ [21]. As for sample S1, M_c_ is at a minimum, N_0_ is at a maximum, and the network structure is optimal. In a sense, the cross-linked network structure of sample S1 is the most regular. Correspondingly, the greater apparent average molecular weight M_c_ benefits the tensile strain [19], and it can be inferred that, for the polytriazole elastomers tested here, when R increases, the elongation tends to increase.

### 2.4. DMA

**Figure 5:**
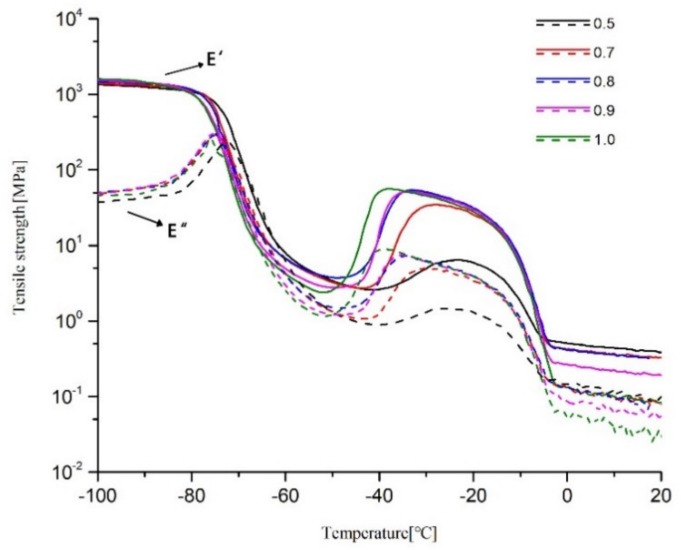
Storage and loss modulus of S1–S5.

**Figure 6:**
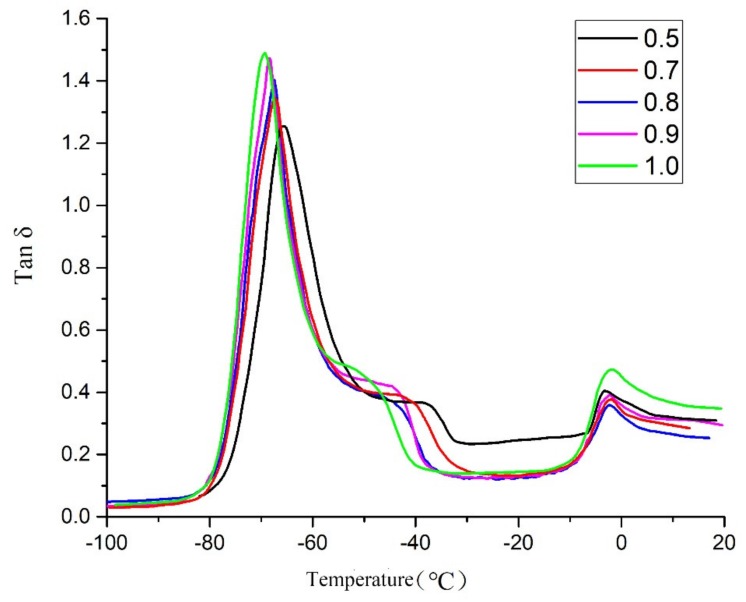
The tan δ curves of S1–S5.

The following parameters were obtained: storage and loss modulus (E′ and E′′ respectively) and loss factor (tan δ = E′′/E′). The temperature of the main peak of the loss factor in DMA is taken to represent Tg. Storage and loss modulus values of five samples were compared in Figure 5, and the loss factors in Figure 6. These figures show that Tg is around −70 to −65 °C. As R increases, Tg decreases slightly because the number of branched chains is reduced. If the number of branched chains is reduced, the time taken from formation to full movement is relatively short and the increased density of cross-linking can also increase Tg, which can also be explained as the larger the R value, the poorer the regularity of the cross-linked network (overall, Tg did not change to any significant extent).

In the temperature range from −50 to −30 °C, the storage modulus and loss modulus increase at the same time, and simultaneously, a small plateau region appears in the tan δ curve in the corresponding temperature range and then decreases. One of the reasons for this phenomenon is the presence of hanging chains [13]. Both tetrahydrofuran homopolymer and ethylene oxide homopolymer are easily crystallized. When the two components are randomly copolymerized, there will be a local high concentration of a certain component, thereby forming partial crystals [22]. The presence of hanging chains, and the probability of a high local concentration of a certain component on the hanging chains mean that, when the temperature rises to a certain value, the copolymer will crystallize locally. Therefore, both storage modulus and loss modulus will increase, and a small platform area will appear in the tan δ curve. As the temperature continues to increase, the crystalline region melts, so the storage modulus and loss modulus decrease, and tan δ decreases. In a polymer, the higher crystallinity leads to a higher melting point. Thus, the larger the R value, the lower the temperature at which the energy storage and the loss modulus begin to change, and the lower the initial temperature of the tan δ plateau region.

The main reasons for this phenomenon are: first, because the various R values are different, the numbers of hanging chains in the samples are also different; second, the crystallinity and crystallization temperature are affected by the degree of cross-linking. When R = 1, specimens contain the largest number of dangling chains, and the degree of cross-linking is low, so the temperature at which it starts to crystallize is the lowest, so it starts to crystallize first. As R decreases, the initial crystallization temperature increases. When the temperature continues to increase, the storage modulus and loss modulus gradually decrease, and this process is the crystalline melting process. Eventually, melting ends at around −5 °C and the process can be seen in Figure 5 and Figure 6.

### 2.5. Thermal Performance

**Figure 7:**
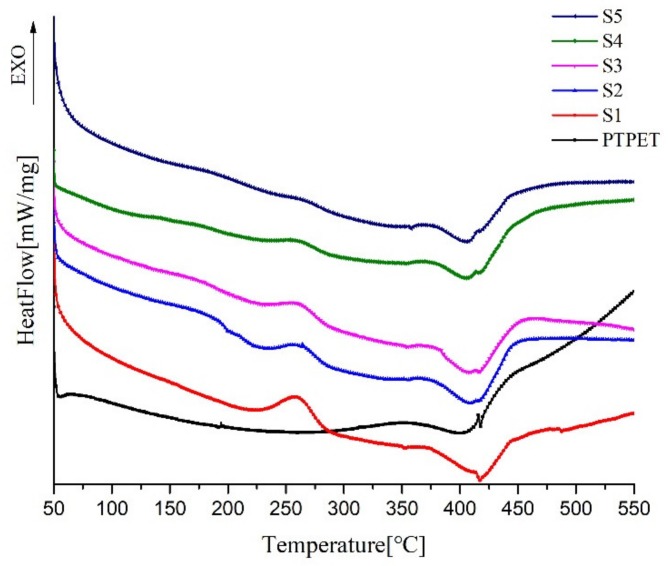
DSC curves of PTPET and S1–S5.

The DSC curves of the five samples are shown in Figure 7. There are several peaks in every curve and temperature of peaks (Table 3). The first typical DSC curve shows the exothermic event with a maximum amplitude at around 258 °C that can be associated with the decomposition of the azide groups to nitrogen molecules. The theoretical calculated values of azide content of the prepared elastomers are listed in Table 4. As R increases, the peak area decreases gradually because the -N_3_ content decreases therewith. When R = 1.0, the peak does not completely disappear, indicating that -N_3_ is not fully involved in the click chemical reaction; thus, when R = 1.0, there should be a small amount of hanging chain inside, which is consistent with the result arising from DMA. The change in the R value does not markedly affect the peak temperature change of the azide group.

Compared to the DSC curve of PTPET, all samples have a slight endothermic peak at temperatures of between 352 and 358 ℃, which is the endothermic peak of the ester-bond cleavage at the upper end of the GAP chain [23].

**Figure 8:**
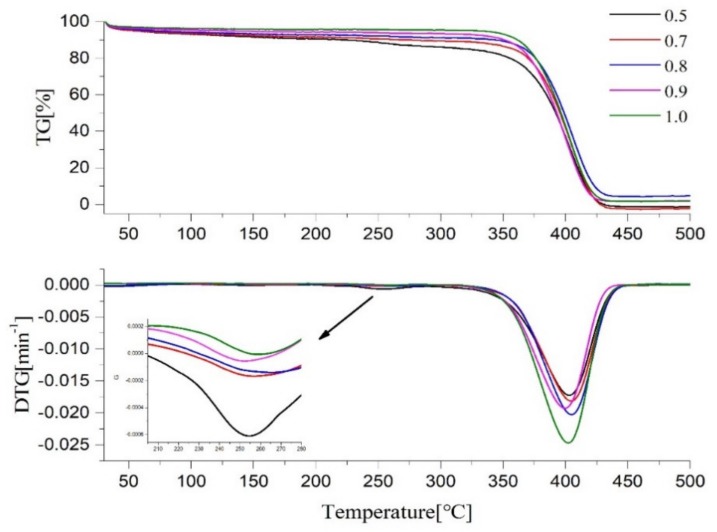
TG curves of PTPET and S1–S5.

The TG/DTG curves of the five samples are shown in Figure 8. Combined with TG/DTG, it can be seen that, when the R value is small, there are two mass loss peaks. The first is the exothermic decomposition of -N_3_ at T = 258 °C, resulting in a mass loss; with the increase of the R value, the mass loss value decreases. When R = 1, the mass loss at this region is not zero, and there is still a slight mass loss peak, demonstrating that the click reaction proceeds (albeit incompletely) and a small amount of hanging chains remain. The second mass loss peak starts at around 350 °C and reaches a maximum at 404 ± 1.5 °C, ending at around 450 °C. The mass loss includes the CH_2_-N bond between the methylene group and the triazole ring being thermally broken; the triazole ring is thermally decomposed to release N_2_, and the break of the ether bond and aliphatic bond occurs in the polymer backbone [24].

As the temperature increased to 500 °C, there remained 5% to 10% mass residue, which suggested that the decomposition of triazole required a higher temperature to decompose [1,2]. This may be because the main chain is surrounded by a triazole ring, and the strong interaction between the molecules creates a so-called “jacket” effect that protects the main chain from heat, thereby improving stability [25]. Therefore, the decomposition temperature of the triazole ring should be in a higher temperature range, indicating that the triazole polyether has strong thermal stability.

## 3. Experimental Work

### 3.1. Materials

The following chemicals were used: low-molecular-weight ester-terminated glycidyl azide polymer (GAP Mn = 800), ethylene oxide-tetrahydrofuran random copolymer (PET, Mn = 4200, functionality = 2) (Luoyang Liming Chemical Research Institute, Luoyang, China); tetrahydrofuran (THF) (AR, Sinopharm Chemical Reagent Co., Ltd., Shanghai, China); potassium tert-butoxide, concentration = 98% (Shanghai Aladdin Biochemical Technology Co., Ltd., Shanghai, China); propargyl bromide, (AR (containing 0.3% MgO), Henan Wanjia Shouhua Biotechnology Co., Ltd., Zhenzhou, China); toluene (AR, Tianjin Shentai Chemical Reagent Co., Ltd., Tianjin, China).

### 3.2. Instrument and Analysis

We performed FTIR using Bruker Vertex 70 instruments; ^1^H NMR was performed using a Bruker Minispec MQ; DSC (DSC 1) and TG (TGA 1) were performed using instruments from Mettler Toledo, Greifensee, Switzerland; dynamic mechanical analysis (DMA 1, Mettler Toledo, Switzerland) was carried out at 1 Hz and a programmed heating rate of 5 K **·** min^−1^ in tension mode (measurements were taken over a temperature range from −100 °C to 20 °C).

### 3.3. Swelling Test

Swelling measurements were carried out in toluene at 20 °C, and the volume fraction of the elastomers was determined gravimetrically. Small pieces of elastomer (about 0.15 g, 10 mm × 5 mm × 3 mm) were immersed in toluene, and the swollen gels were removed from the solvent at different times, quickly blotted with dry filter paper, and weighed until the mass change was less than 0.01 g. 

### 3.4. Preparation of PTPET

We dissolved 20 g of PET in 100 mL of THF in a single-port flask, and 1.4 g of potassium t-butoxide was weighed and dissolved in 50 mL of THF. The reaction flask was placed in an ice bath for half an hour, then 1.2 mL of propyne bromide was added dropwise to the flask, while being agitated on a magnetic stirrer, then placed in a water bath at 30 °C, magnetically stirred, condensed, and refluxed for 48 h. Afterwards, we washed the resulting mixture with saturated brine and deionized water: firstly at 75:25, secondly at 50:50, then at 100:0 before dispensing the fluid through a separating funnel. Finally, the organic layer thus obtained was placed in an oven at 50 °C until the THF was completely removed, after which a light golden liquid was obtained.

### 3.5. Curing of PTPET with GAP

**Figure 9:**
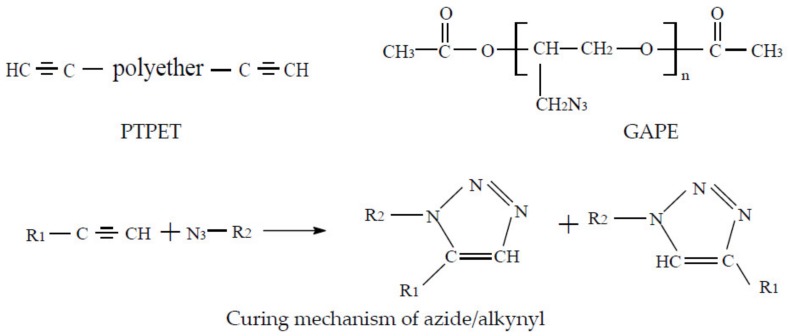
The reaction mechanism of azide and alkynyl.

The R value is the equivalent ratio of the prepolymer functional group and the cross-linker functional group (R = [alkyne]/[azide]). According to the requirement of the R value, PTPET and GAP were weighed and mixed by stirring at room temperature until a homogeneous mixture was formed. The schematic diagram of the chemical reaction is shown in Figure 9. The curing process was free of copper. The mixtures were poured into molds, then placed overnight at normal temperature and pressure to discharge bubbles. Finally, we put the molds into an oven for 7 days at 60 °C. The theoretical values of azide content of the polytriazole elastomers and GAP are listed in Table 4.

## 4. Conclusions

The terminal hydroxyl group PET was modified to a propargyl group by functional group modification and the desired PTPET was obtained. The results of FTIR and ^1^H NMR proved that the product was PTPET. The PTPET and GAP were cured without the use of a catalyst by using a click reaction for 7 d at 60 °C to obtain the samples. Through tensile testing, when R = 0.5, the tensile strength could reach 0.332 MPa and elongation at break was 897.1%. After that, the tensile strength and elongation of the samples decreased as R increased. The results of DMA show that the Tg of samples were around −70 to −65 °C, and a small amount of crystallization appeared between −50 and −30 °C. Through DSC and TG experiments, when R was small, the decomposition peak of -N_3_ was found at 258 °C, the main mass loss started at 350 °C and reached its maximum at about 404 °C, mainly as a result of the breaking of the ether bond and aliphatic bond in the polymer backbone. When the temperature reached 500 °C, there remained 5% to 10% mass residue, which suggests that the decomposition of triazole required a higher temperature. Therefore, the thermal properties of the polytriazole elastomer were stable.

In general, the elastomer has good thermal stability, excellent elongation, and low glass transition temperature. It can be considered for use as a rocket composite propellant binder.

## Figures and Tables

**Table 1:** Mechanical properties of polytriazole elastomers.

Sample	R Value	Tensile Strength, σ_b_/MPa	Elongation, ε_b_/%
S0	0.3	0.262	808.9
S1	0.5	0.332	897.1
S2	0.7	0.312	258.7
S3	0.8	0.3085	250.3
S4	0.9	0.305	327.1
S5	1.0	0.277	410.2

**Table 2:** Structural parameters of PTPET-based elastomers.

Sample.	R Value	χ_1_	Q_v_	V_2m_	ρ g · cm^−3^	Mc g · mol^−1^	N_0_ mmol · cm^−3^
S1	0.5	0.383	10.996	0.091	1.029	7201.1	0.143
S2	0.7		11.340	0.088	1.091	7925.1	0.138
S3	0.8		12.348	0.081	1.048	8449.7	0.124
S4	0.9		13.136	0.076	1.109	9630.4	0.115
S5	1.0		14.581	0.069	1.059	10416.5	0.102

**Table 3:** Peak temperature of DSC curves.

Samples	1st Peak/℃	2nd Peak/℃	3rd Peak/℃
PTPET	--	--	416.0
S1	258.4	352.7	414.0
S2	258.8	355.4	413.7
S3	259.2	354.0	414.0
S4	259.4	353.1	414.0
S5	260.5	358.0	414.8

**Table 4:** The theoretical values of azide content of the polytriazole elastomers.

Sample	Elastomers					GAP
	R = 0.3	R = 0.5	R = 0.7	R = 0.8	R = 1.0	
Azide content (by weight)	4.0%	1.9%	0.9%	0.5%	0	37.0%
